# Association between physical activity and sleep quality among healthcare students

**DOI:** 10.3389/fspor.2024.1357043

**Published:** 2024-02-14

**Authors:** MinatAllah Alhusami, Noora Jatan, Skylar Dsouza, Meshal A. Sultan

**Affiliations:** College of Medicine, Mohammed Bin Rashid University of Medicine and Health Sciences, Dubai, United Arab Emirates

**Keywords:** physical activity, exercise, medical students, nursing students, dental students, biomedical sciences students, sleep quality

## Abstract

**Objective:**

To assess the relationship between physical activity (PA) and sleep quality (SQ) in Mohammed Bin Rashid University (MBRU) students in Dubai, United Arab Emirates. Taking into account this being one of the first studies exploring this aspect among healthcare students in the Gulf Cooperation Council (GCC) countries.

**Methods:**

This is an analytical cross-sectional study that involved disseminating online surveys via email to students of all 5 colleges in MBRU between March-June 2023. The survey encompassed queries on demographics, the Saltin-Grimby Physical Activity Level Scale (SGPALS), the International Physical Activity Questionnaire-Short Form (IPAQ-SF), and the Pittsburgh Sleep Quality Index (PSQI).

**Results:**

The survey was completed by 105 students. Most students were from the medical college (98/105; 93.3%) and were females (84/105; 80.0%). Females engaged the most in low PA (44.0%) while males engaged the most in high PA (42.9%) according to the IPAQ-SF. There was a significant association between SGPALS and gender (*p* = 0.007, *X*^2^*^ ^*= 12.0). The global PSQI score showed that 70.5% of the students had bad-quality sleep. Sleep efficiency and leisurely PA are significantly negatively correlated (*p* = 0.026, *ρ* = −0.217) while sitting minutes and sleep duration are significantly positively correlated (*p* = 0.030, r = 0.212).

**Conclusion:**

Significant negative correlations between sleep efficiency and leisurely PA demonstrate that while exercise has been implicated in the improvement of SQ, excessive exercise can behave as an additional stressor and induce negative changes in the SQ of healthcare students. Meanwhile, significant positive correlations between sleep duration and sitting indicate that adequate rest should not be undervalued in its effects on sleep. Furthermore, the findings of this study highlight public health implications that warrant attention by educators and policymakers in academic health systems.

## 1 Introduction

Understanding the intricate relationship between physical activity and sleep quality is paramount, especially when considering the unique challenges faced by healthcare students. This demographic probably encounters heightened stress levels due to the rigorous demands of their academic and clinical responsibilities (1). The association between physical activity and sleep quality is of particular significance in this context, as these students likely navigate a delicate balance between maintaining their well-being and excelling in their professional pursuits.

Across all life domains, sleep remains an intrinsic biological feature that plays a significant part in several “somatic, cognitive, and psychological processes” (2). As such, altered sleep quality (SQ) can have detrimental effects on body homeostasis (2). Aside from pharmacological intervention, physical activity (PA) has been highlighted in literature for its potential to improve SQ, the mechanisms of which have been explored extensively. The release of pro-inflammatory cytokines Interleukin-1 and -6, as well as Tumor necrosis factor-alpha can induce feelings of drowsiness during moderate-intensity exercise (3). Furthermore, brain-derived neurotrophic factor release increases after exercise, and it enhances “memory processing during sleep” (4, 5). Lastly, the most “potent physiologic stimulus” for growth hormone secretion is PA (6). Growth hormone secretion is associated with slow-wave sleep also known as deep sleep, thus illustrating another link between PA and SQ (7).

In a 2011 randomized controlled trial in South Carolina, a 12-week regimen of moderate-intensity aerobic and resistance exercise for 43 obese/overweight participants (18–55 years) resulted in a 19% reduction in poor-quality sleep, as assessed by the Pittsburgh Sleep Quality Index (PSQI) post-exercise training (8). A 2020 randomized controlled trial in Saudi Arabia, involving 80 chronic primary insomnia patients (35–56 years), demonstrated significant improvements (*p* < 0.05) in sleep efficiency (measured by polysomnography) and self-esteem (measured by the Rosenberg Self-Esteem Scale) in the group that underwent six months of supervised aerobic exercise (9).

Despite its importance, sleep remains to be a persistent issue among students. In a 2021 cross-sectional study based in the United Arab Emirates (UAE), with a sample of 96 medical and dental students (mean age 19.5 and 29.3 respectively), 84.3% reported PSQI scores suggestive of poor-quality sleep (10). Similarly, a 2022 cross-sectional study based in Iran with 138 dental students of 21–30 years of age, reported a significant relationship (*p* = 0.002) as per the chi-squared test between high Global PSQI scores (poor SQ) and high scores on the Dental Environment Stress questionnaire (11). This suggests that Environmental stress negatively impacts SQ (11).

Poor SQ is a prevalent issue among healthcare students, driven by academic demands (12). A 2012 study in India involving 30 medical undergraduates (aged 18–25) revealed that performing tasks requiring sustained attention five times over 24 h without sleep significantly increased errors in two out of three tests (*p* < 0.001), suggesting that sleep deprivation impacted the students' judgment ability (13). Despite the importance of SQ, there is a lack of research on its correlation with PA in students across multiple healthcare colleges. This study addresses that gap in the GCC region, focusing medical, dental, nursing, and biomedical Doctor of Philosophy (PhD.) and Master of Science (MSc.) students.

### 1.1 Aim

This study aims to identify whether PA can serve as a non-pharmacological way of improving SQ among students of all degrees.

### 1.2 Objectives

(1) To describe the characteristics of PA and SQ in MBRU students.
(2) To identify gender-related and college-related differences (medical/dental/nursing/biomedical MSc. & PhD.) in PA and SQ students at MBRU.
(3) To assess individual components of SQ and compare them to various levels and components of PA.
(4) To investigate whether the relationship between PA and SQ within MBRU students is comparable to healthcare students in other universities as well as to other university majors.

## 2 Materials and methods

### 2.1 Study design, setting, and participants

This study is an observational analytical CS study involving medical, dental, nursing, and biomedical sciences PhD. and MSc. students enrolled at the Mohammed Bin Rashid University of Medicine and Health Sciences (MBRU) in the year 2023. This study was carried out in Dubai, UAE, spanning a duration of 11 months, with data collection running from March to June 2023 and final data analysis completed in September 2023. Adherence to the STROBE guidelines was maintained in the reporting of this study (14).

Inclusion criteria for this study encompassed students in the medical, dental, nursing, and biomedical sciences (MSc., PhD.) programs at MBRU in the year 2023, and no exclusion criteria were applied during the recruitment process.

### 2.2 Variables

This study examines the effect of exercise duration, intensity, modality, time of day, and frequency on characteristics of SQ while analyzing differences in outcome data by age, gender, nationality, and college (demographics).

### 2.3 Data sources/measurement

The data collection tool consisted of a Google Forms survey sent out to all MBRU students from the colleges of medicine, dentistry, nursing, and biomedical sciences (MSc., PhD.) by email. The survey collected demographic details, followed by three standardized questionnaires assessing exposure and outcome data.

The first questionnaire was the Saltin–Grimby Physical Activity Level Scale (SGPALS), a 4-level self-rated questionnaire assessing leisurely PA over the past year; the lowest tier being “physically inactive”, and the highest tier being “regular hard physical training for competitive sports” (15).

To assess exercise duration, frequency, and intensity, the survey included the International Physical Activity Questionnaire-Short Form (IPAQ-SF), a 7-item self-rated questionnaire that records the amount of time spent engaging in vigorous-intensity PA, moderate-intensity PA, walking, and sitting in the past seven days (16). The survey also included additional questions (not within the standardized questionnaires) inquiring about students' preferred exercise modality and time of day.

The Arabic short version of the IPAQ has established acceptable validity and reliability among Arabic population (17). It was also used in studies involving Arabic students (18). However, since the students at MBRU are from multiple nationalities and English is the teaching language we used the original IPAQ-SF (16).

The Pittsburgh Sleep Quality Index (PSQI) was included last. It is a 19-item self-rated questionnaire that examines SQ based on 7 component scores: subjective SQ, sleep latency, sleep duration, sleep efficiency, sleep disturbance, sleep medication use, and daytime dysfunction, respectively; these did not include items from question 10. Each component yields a score of 0–3, with 0 indicating the highest quality of sleep and 3 indicating the greatest dysfunction (19, 20).

The consent form and survey are available on: https://docs.google.com/document/d/1JPLxsRbzqpWCx2PvAOQdoAq53kjMEf8bdZkjp-3cAUc/edit?usp=sharing

### 2.4 Study size

**Figure 1:**
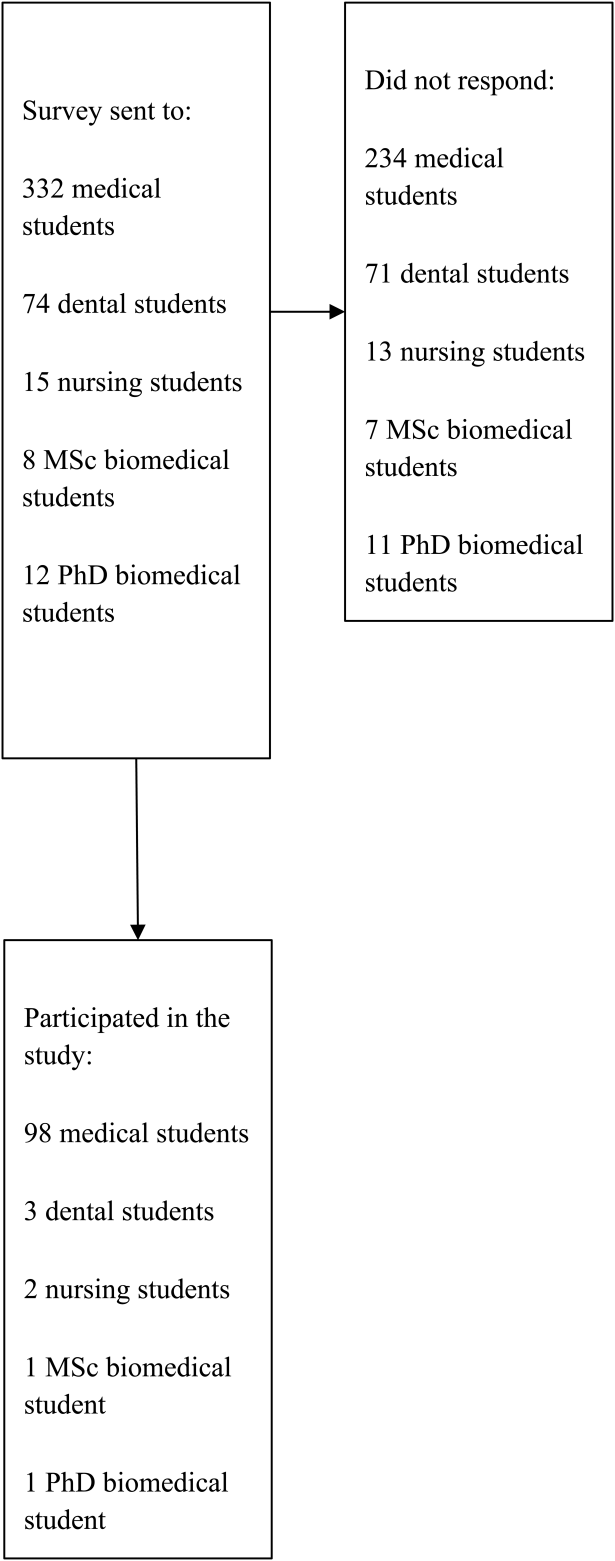
Flow chart of participants.

All volunteering MBRU students were eligible to participate in the study. Thus, no sample size was statistically calculated as it was based on obtaining complete coverage. The final sample size was 105 students (Figure 1).

### 2.5 Quantitative variables

The IPAQ-SF provided the sum of days and minutes spent engaging in vigorous PA, moderate PA, and walking, along with the number of minutes spent sitting on a weekday. The sum of all these represented each PA modality in MET-minutes/week: MET-level × min of activity/day × days/week. This was done thrice, yielding separate total MET-minutes/week for each modality (walking = 3.3 METs, moderate = 4.0 METs, vigorous = 8.0 METs). The sum of these produced total MET-min/week for each student in the sample. Hereafter, “MET-walking”, “MET-moderate”, and “MET-vigorous” refers to MET-min/week spent walking, engaging in moderate PA, and engaging in vigorous PA, respectively.

PSQI components yielding continuous data included sleep latency, sleep duration, sleep efficiency, sleep disturbance, and daytime dysfunction. The sum of all PSQI component scores generated a global score of 0–21.

### 2.6 Qualitative variables

Each PSQI component score represented an individual categorical variable ranging from 0 to 3. Global PSQI scores were grouped into “good SQ” (0–5) and “poor SQ” (6–21). IPAQ-SF data generated scores of “low”, “moderate”, or “high” PA (18). PA time of day was categorized as “morning” (5:00am–11:59am), “afternoon” (12:00pm–4:59pm), “evening” (5:00pm–8:59pm), and “night” (9:00pm–4:59am), and PA modality as “aerobic”, “anaerobic”, “muscular”, and “flexibility”. The demographic data included gender (male/female), nationality (UAE-national/expatriate), age (≤18/19–20/≥20), and college (medicine/dentistry/nursing/biomedical PhD./biomedical MSc).

### 2.7 Statistical methods

Statistical analysis was conducted on the Statistical Package for Social Sciences (SPSS), version 28. Statistical significance was set at *p* ≤ 0.05. To measure the strength and direction of a relationship between two continuous variables, Pearson's correlation coefficient (*r*) test was used; between a continuous and a categorical variable, Spearman's rank correlation coefficient (*ρ*) test was used—both yielding numbers between −1 and +1 (21). Lastly, Pearson's chi-square (*X*^2^) test measured the association between two categorical variables with a minimum chi-square value set at *X*^2^ = 3.84 (22). The choice of these tests was based on their suitability for detecting associations between such variables.

## 3 Results

Table 1 displays students' demographics with mean age. A total of 105 students completed the survey, of which, 20% were males and 80% were females. The mean age (SD) of males was 19.4 (1.0) and females was 21.0 (3.7). 39% were Emiratis and 60% were expatriates, with a mean age (SD) of 20.4 (3.7) for Emiratis and 20.8 (3.2) for expatriates. Nearly 93% of participants were from the college of medicine, 3% from dentistry, 2% from nursing, 1% from biomedical sciences MSc. and 1% from biomedical PhD. Medical students' mean age was 20.0 (2.0), dental was 30.0 (1.0), nursing was 28.5 (6.4), biomedical MSc. was 38.0, and biomedical PhD. was 22.0.

**Table 1:** Student demographics with mean age.

	*N* (%)	Mean age (years)	SD
Gender			
Male Female	21 (20.0) 84 (80.0)	19.4 21.0	1.0 3.7
Nationality			
Emirati Others	41 (39.0) 64 (61.0)	20.4 20.8	3.7 3.2
College			
Medicine Dentistry Nursing Biomedical MSc. Biomedical PhD.	98 (93.3) 3 (2.9) 2 (1.9) 1 (1.0) 1 (1.0)	20.0 30.0 28.5 38.0 22.0	2.0 1.0 6.4 0.0 0.0

*N*, number, %, percentage; SD, standard deviation.

Table 2 displays the differences in PA and SQ by gender. According to IPAQ-SF, majority of the study sample engaged in low PA (40.0%), followed by high PA (31.4%), and then moderate PA (28.6%). This means that 40.0% of the students (of which over a fifth of males and majority of females) did not participate in a sufficient PA to satisfy public health recommendations—at least 5 or more days of moderate PA a week—thus, are considered inactive based on IPAQ-SF (23). Most females preferred low PA (44.0%), while most males preferred high PA (42.9%). There was a significant association between SGPALS and gender (*p* = 0.007, *X*^2^*^ ^*= 12.0). SGPALS showed that nearly 50% of students did light PA, followed by 23.8% of the students either being inactive or doing regular PA, and nearly 5% doing regular hard PA. Majority of females engaged in light PA (52.4%), while majority of males engaged in regular PA (52.4%). Both genders engaged least in regular hard PA (females *N* = 4, males *N* = 1). The global PSQI score revealed nearly three-quarters of the sample had poor SQ.

**Table 2:** Physical activity (IPAQ-SF and SGPALS) and sleep quality (global PSQI) based on gender.

** **		Overall	Gender		** **	** **
** **		** **	Male	Female	*p*-value	*X* ^2^
** **		*N* (%)	*N* (%)	*N* (%)	** **	** **
IPAQ-SF	Low	42 (40.0)	5 (23.8)	37 (44.0)		
** **	Moderate	30 (28.6)	7 (33.3)	23 (27.4)	0.221	3.019
** **	High	33 (31.4)	9 (42.9)	24 (28.6)		
SGPALS	Inactive	25 (23.8)	3 (14.3)	22 (26.2)		
** **	Light PA	50 (47.6)	6 (28.6)	44 (52.4)	0.007	12.000
** **	Regular PA	25 (23.8)	11 (52.4)	14 (16.7)		
** **	Regular hard PA	5 (4.8)	1 (4.8)	4 (4.8)		
Global PSQI	Good quality sleep	31 (29.5)	7 (33.3)	24 (28.6)	0.669	0.183
** **	Bad quality sleep	74 (70.5)	14 (66.7)	60 (71.4)		

*N*, number; %, percentage by gender.

Table 3 displays the differences in PA and SQ by college. Medical students mostly engaged in low PA (36.7%), followed by high PA (32.7%), followed by moderate PA (30.6%). All of those who did moderate PA were medical students (30.6%). With SGPALS, nearly half the medical students did light PA and engaged least in regular hard PA (5.1%), with an equal spread between inactivity and regular PA (both 23.5%). Twice as many dental students did regular PA compared to light PA (33.3%). All of the nursing students did light PA, while all of the biomedical MSc. and PhD. students remained inactive. The global PSQI score reveals that the majority of students within each college had poor SQ.

**Table 3:** Physical activity (IPAQ-SF and SGPALS) and sleep quality (global PSQI) based on colleges.

		Overall			Colleges				
			Medicine	Dentistry	Nursing	Biomedical PhD.	Biomedical MSc	*p-*value	*X* ^2^
		*N* (%)	*N* (%)	*N* (%)	*N* (%)	*N* (%)	*N* (%)		
IPAQ-SF	Low	42 (40.0)	36 (36.7)	2 (66.7)	2 (100.0)	1 (100.0)	1 (100.0)		
	Moderate	30 (28.6)	30 (30.6)	0 (0.0)	0 (0.0)	0 (0.0)	0 (0.0)	0.449	7.845
	High	33 (31.4)	32 (32.7)	1 (33.3)	0 (0.0)	0 (0.0)	0 (0.0)		
SGPALS	Inactive	25 (23.8)	23 (23.5)	0 (0.0)	0 (0.0)	1 (100.0)	1 (100.0)		
	Light PA	50 (47.6)	47 (48.0)	1 (33.3)	2 (100.0)	0 (0.0)	0 (0.0)	0.451	11.936
	Regular PA	25 (23.8)	23 (23.5)	2 (66.7)	0 (0.0)	0 (0.0)	0 (0.0)		
	Regular hard PA	5 (4.8)	5 (5.1)	0 (0.0)	0 (0.0)	0 (0.0)	0 (0.0)		
Global PSQI	Good quality sleep	31 (29.5)	30 (30.6)	1 (33.3)	0 (0.0)	0 (0.0)	0 (0.0)	0.781	1.752
	Bad quality sleep	74 (70.5)	68 (69.4)	2 (66.7)	2 (100.0)	1 (100.0)	1 (100.0)		

*N*: number, %: percentage by college.

Table 4 displays the differences in PA and SQ by age. According to IPAQ-SF, of those under 18, 39.1% engaged in moderate PA and 26.1% engaged in high PA, while from ages 19–20, 40.9% engaged in low PA and 22.7% engaged in moderate PA; lastly, 43.2% above age 21 engaged in low PA and 27.0% engaged in moderate PA. Majority of the students engaged in light PA while the least engaged in regular hard PA across all 3 age groups according to SGPALS. The global PSQI scores reveal that around two-thirds of the students within each of the age groups have poor SQ.

**Table 4:** Physical activity (IPAQ-SF and SGPALS) and sleep quality (global PSQI) based on age.

** **		Overall	Age (years)		** **	** **	** **
** **		** **	≤18	19–20	≥21	*p*-value	*X* ^2^
** **		*N* (%)	*N* (%)	*N* (%)	*N* (%)	** **	** **
IPAQ-SF	Low	42 (40.0)	8 (34.8)	18 (40.9)	16 (43.2)		
** **	Moderate	30 (28.6)	9 (39.1)	10 (22.7)	10 (27.0)	0.680	2.304
** **	High	33 (31.4)	6 (26.1)	16 (36.4)	11 (29.7)		
SGPALS	Inactive	25 (23.8)	6 (26.1)	10 (22.7)	9 (24.3)		
** **	Light PA	50 (47.6)	11 (47.8)	18 (40.9)	20 (54.1)	0.701	3.818
** **	Regular PA	25 (23.8)	4 (17.4)	14 (31.8)	7 (18.9)		
** **	Regular hard PA	5 (4.8)	2 (8.7)	2 (4.5)	1 (2.7)		
Global PSQI	Good quality sleep	31 (29.5)	9 (39.1)	13 (29.5)	9 (24.3)	0.475	1.489
** **	Bad quality sleep	74 (70.5)	14 (60.9)	31 (70.5)	28 (75.7)		

*N*, number; %, percentage by age.

Table 5 demonstrates a significant negative correlation between sleep efficiency (PSQI) and SGPALS (*p* = 0.026, *ρ* = −0.217).

**Table 5:** Sleep efficiency (PSQI component 4) compared to SGPALS.

** **	** **	SGPALS
Sleep efficiency	Correlation coefficient	−0.217
** **	*p-*value	0.026

Tables 6, 7 compare daytime dysfunction (PSQI) with MET-walking (IPAQ-SF), item “cannot breathe comfortably” (PSQI item 5d), sleep disturbance (PSQI), and number of days of vigorous activity (IPAQ-SF item 1). There is a significant positive correlation between daytime dysfunction and MET-walking (*p* = 0.007, *r* = 0.260), item “cannot breathe comfortably” (*p* = 0.006, *ρ* = 0.268), sleep disturbance (*p* < 0.001, *r* = 0.485), and a significant negative correlation with number of days of vigorous activity (*p* = 0.040, *r* = −0.201).

**Table 6:** Daytime dysfunction (PSQI component 7) compared to MET-walking (IPAQ-SF) and number of days of vigorous activity (IPAQ-SF component 1).

** **	** **	MET-walking (IPAQ-SF)	Number of days of vigorous activity (IPAQ-SF1)
Daytime dysfunction	Correlation coefficient	0.260	−0.201
** **	*p-*value	0.007	0.040

**Table 7:** Daytime dysfunction (PSQI component 7) compared to cannot breathe comfortably (PSQI competent 5d) and sleep disturbance (PSQI component 5).

** **	** **	Cannot breathe comfortably (PSQI5d)	Sleep disturbance (PSQI component 5)
Daytime dysfunction	Correlation coefficient	0.268	0.485
** **	*p-*value	0.006	<0.001

Table 8 compares global PSQI to subjective sleep quality (PSQI component 1), total MET-min/week (IPAQ-SF), and the variable exercise time of day. There is a significant association between the global PSQI score and subjective SQ (*p* < 0.001, *X*^2 ^= 18.775), a non-significant negative correlation with total MET-min/week (*p* = 0.751, *ρ* = −0.031), and a non-significant association with “exercise time of day” (*p* = 0.950, *X*^2 ^= 0.352).

**Table 8:** Global PSQI compared to different variables.

** **	** **	Subjective sleep quality (PSQI component 1)	Total MET-min/wk (IPAQ-SF)	Exercise time of day
Global PSQI	Correlation coefficient *X*^2^ *p* value	– 18.775 <0.001	−0.031 – 0.751	– 0.352 0.950

Table 9 compares subjective SQ (PSQI component 1) to global PSQI. Additionally, 74 students had poor SQ as per PSQI score, and of those, 5.4% claimed to have “very good” SQ and 59.5% “fairly good” SQ—that is 64.9% of students with poor SQ *self*-*reported* good SQ. Of the 31 students who had good SQ.

**Table 9:** Subjective SQ (PSQI component 1) compared to global PSQI.

		Global PSQI Good sleep quality *N* (%)	Poor sleep quality *N* (%)	Total *N* (%)
Subjective sleep quality Total [*N* (%)]	Very good Fairly good Fairly bad Very bad	9 (29.0) 21 (67.7) 1 (3.2) 0 (0.0) 31 (100.0)	4 (5.4) 44 (59.5) 22 (29.7) 4 (5.4) 74 (100.0)	13 (12.4) 65 (61.9) 23 (21.9) 4 (3.8) 105 (100.0)

Table 10 compares sitting minutes (IPAQ-SF item 7) with hours of sleep (PSQI item 4) and sleep efficiency (PSQI component 4). There is a significant positive correlation between sitting minutes and sleep duration (*p* = 0.030, *r* = 0.212) and a significant negative correlation with sleep efficiency (*p* = 0.003, *ρ* = −0.287).

**Table 10:** Sitting min (IPAQ-SF7) compared to different sleep variables.

** **	** **	Sleep duration (PSQI4)	Sleep efficiency (PSQI component 4)
Sitting minutes	Correlation coefficient	0.212	−0.287
** **	*p-*value	0.030	0.003

Table 11 compares total MET-min/week to all colleges and shows a significant negative correlation between the two (*p* = 0.020, *ρ* = −0.227).

**Table 11:** Total MET-min/week (IPAQ-SF) compared to all colleges.

		Total MET-min/wk
Colleges	Correlation coefficient	−0.227
	*p-*value	0.020

## 4 Discussion

This study revealed patterns between various PA characteristics and SQ components. In terms of PA intensity, students who preferred walking over vigorous PA faced difficulty staying awake during the day and/or had reduced enthusiasm for daily tasks. Those exhibiting such daytime dysfunction reported increased sleep disturbance, namely difficulty breathing at night. It is difficult to tell whether said effects are a result of inactivity among these students or if fatigue from their daytime sleepiness and associated symptoms rendered them unable to engage in vigorous PA, thus they preferred to walk.

That being said, students with longer sitting minutes during work, study hours, or leisure, reported longer sleep hours. One explanation for this is that long hours spent sitting to study/work can increase fatigue and thus the demand for longer sleep duration. This is further supported by the finding that those pursuing postgraduate studies had significantly less total MET-min/week, indicating that healthcare students become progressively inactive with increasing workload. Meanwhile, in a 2009 prospective cohort study in the United Kingdom on British civil servants (*n* = 2,436–2,459) aged 35 and above, longer work hours led to self-reports of reduced sleep duration (<7 h) in 59.8% of the participants; perhaps said differences are due to the entirely different stressors that they face given the nature of their work (24).

Another plausible explanation is that high leisurely sitting time can be a form of sedentary behavior and, therefore, more likely to be observed in someone with longer sleep hours, however, this hypothesis was not supported by any other findings in this study. In a 2016 CS study conducted in “five urban regions across Europe” with a study sample of mean age of 51.9 years (*n* = 6,037), more sedentary behavior (defined as time spent sitting assessed by the Marshall Questionnaire) yielded shorter hours of sleep (*p* < 0.05) (25). All in all, it seems that sitting results in a longer duration of sleep in healthcare students but not necessarily in other population types.

Students with high PA by SGPALS level and prolonged sitting exhibited decreased sleep efficiency. Intense PA may disrupt sleep efficiency through hypothalamic-adrenal axis dysregulation, as observed in insomnia cases (26). A 2008 North Carolina study on 12 moderately trained men revealed significantly elevated cortisol levels after 30 min of PA at 60%, or 80% intensity, of their maximum oxygen consumption, while cortisol decreased significantly after 30 min of sitting (27). Increased cortisol can lead to sleep deprivation, perpetuating a cycle of constant sleep deterioration (26). However, a systematic review suggested that a balanced, long-term low-to-moderate PA regimen reduces cortisol levels and improves PSQI scores, emphasizing the importance of moderation to avoid potential adverse effects (28).

Students' PA levels were linked to individual components of SQ, but this study saw no significant correlation between total MET-min/week and global PSQI scores. This suggests each group faced drastically dissimilar challenges regarding sleep hygiene, which were masked by other components added to their global PSQI scores. Essentially, this study saw an indirect link between PA and SQ.

These findings are dissimilar to past literature that saw a more direct relationship between PA and SQ in both healthcare students from other institutions and students from pooled colleges. For one, in a 2021 CS study surveying 96 medical students aged 17–24 using the Global Physical Activity Questionnaire and PSQI at Tarumanagara University, Indonesia, 42 students had poor SQ (global PSQI ≥ 6) with low PA level (<600 MET-min/week), and 22 students had good SQ (global PSQI < 6) with high PA level (≥600 MET-min/week) (*p* = 0.003) (29). In 2022, a CS online survey using the Physical Activity Rating Scale-3 and PSQI was completed by 5,075 students from 3 universities in China (grades 1–4) and found that the PSQI score was significantly negatively correlated with PA level (*p* < 0.001, *r* = −0.159) (30).

### 4.1 Implications for public health educators

Given the significant negative correlation between high-intensity leisurely PA and sleep efficiency, it is imperative to promote PA in moderation to healthcare students as, clearly, excessive exercise can negatively impact overall SQ. Likewise, the significant positive correlation between sitting minutes and hours of sleep highlights the need for healthcare institutions to promote more study breaks. Arguably the most important implication of this study is to educate healthcare students on what constitutes good quality sleep. Clearly, there was a mismatch between participants' perceived SQ and global PSQI score: 64.9% of participants self-reporting good and fairly good SQ in PSQI Component 1 had global PSQI scores indicative of poor SQ. This highlights healthcare students' poor comprehension of sleep hygiene which can be corrected through implementing seminars on SQ.

Furthermore, this study offers crucial insights into the intricate connection between physical activity and sleep quality specifically within the healthcare student demographic. The unique patterns observed in this population, which is often under stress, calls for careful considerations by educators and policymakers in the healthcare education sector. Implementation of various initiatives can be explored, for instance structured physical exercise activities in health colleges to improve sleep quality.

### 4.2 Strengths, limitations, & generalizability

As for study strengths, the use of a unique sample population including students from various colleges including medical, dental, nursing, biomedical PhD., and biomedical MSc. provides a better representation of sleep and exercise trends among healthcare students. Moreover, comparisons of such trends with previous literature were facilitated using standardized questionnaires like SGPALS, PSQI, and IPAQ-SF.

Confounders like caffeine intake, tobacco smoking, academic workload, and other stresses were not adjusted for, and thus possibly influenced SQ alongside PA. Association between smoking, caffeine consumption, and insomnia was highlighted in a previous study on medical students (31). Furthermore, a systematic review highlighted the negative impact of high workload on sleep quality of healthcare students (32). Therefore, it is important for future research to explore the impact of these factors on sleep and physical activity among healthcare students in the GCC region in more depth.

Moving on, another limitation pertains to IPAQ-SF as it asks about PA in *the last seven days*. Unexpected circumstances or temporal academic demands could have led to changes in PA habits in that week for any given participant; hence the Global Physical Activity Questionnaire (GPAQ) could be a better alternative as it asks about PA in a “typical week” (33). Recall bias is common in the context of recalling past experiences (34). Studies have indicated that multiple factors contribute to this form of bias, while higher level of education was associated with more accurate recall (35). Tools utilized in this study, IPAQ-SF, PSQI, and SGPALS elicit the recall of information from variable points in time, which serves as a source of recall bias in this study. This bias can be eliminated through future cohort/ longitudinal studies that assess PA and SQ throughout healthcare students' university duration.

Furthermore, there was a greater number of participants from the college of medicine (*n* = 98) compared to dentistry (*n* = 3), nursing (*n* = 2), biomedical PhD. (*n* = 1), and biomedical MSc. (*n* = 1). This can affect the internal validity of the study since the results are more representative of and therefore skewed in the favor of medical students. Employing strategies utilizing multiple communication methods, for instance, phone calls and mobile messages can enhance participation from other colleges to ensure better data representation. Students at MBRU come from various nationalities. However, since the sample is derived from only one university, it is likely not generalizable to other healthcare students or the general population, which demands including healthcare students from multiple universities for future studies. Lastly, given that 64.9% of participants reported “very good” and “fairly good” SQ in PSQI component 1 had high global PSQI scores (meaning poor quality sleep), this component should be separated from the global PSQI score to eliminate the effect of subjective self-report on objectified SQ score.

### 4.3 Areas for future research

To clarify the temporal relationship between SQ and PA, longitudinal/cohort studies need to be conducted on healthcare students. Future studies should also investigate the positive correlation between sitting minutes and sleep duration among healthcare students and not the general population—such a study can perhaps uncover unique confounders that affect sleep only in healthcare students. Similarly, investigations must be done to identify reasons for the variability between healthcare students' responses to exercise and sleep by controlling for potential confounders like caffeinated beverages/supplements, nicotine products, alcohol, and comorbid physical/mental disorders.

## 5 Conclusion

Despite the plethora of research on sleep and PA among healthcare students, this study is seemingly a first in analyzing said variables on healthcare students of not just one but all colleges (medical, dental, nursing, biomedical PhD., and biomedical MSc.), while also representing both UAE-national and expatriate healthcare students in MBRU, Dubai. The study findings on reduced sleep efficiency with leisurely PA and increased sleep duration with greater sitting minutes indicate the need for system-wide changes in the way that both rest and PA are promoted among healthcare students. Institution-led advocacy for avoiding excessive exercise and encouraging rest can bring about major changes in sleep hygiene among this unique population. Additionally, future research based on longitudinal designs to assess changes in sleep patterns over time as well as exploring innovative interventions aimed at improving sleep quality could provide valuable directions for the future.

## Data Availability

The original contributions presented in the study are included in the article/Supplementary Material, further inquiries can be directed to the corresponding author.
